# Facilitators and barriers in interprofessional collaboration around physical activity on prescription—a focus group study in a Swedish school setting

**DOI:** 10.3389/fspor.2024.1431786

**Published:** 2024-07-17

**Authors:** Emelie Wiklund, Jenny Vikman, Maria Wiklund, Susanna Hedenborg

**Affiliations:** ^1^ Department of Sports Sciences, Malmö University, Malmö, Sweden; ^2^ Physiotherapy Unit, Department of Community Medicine and Rehabilitation, Umeå University, Umeå, Sweden

**Keywords:** children, health promotion, interprofessional collaboration, physical activity, school, school health service

## Abstract

In Swedish school health services, local initiatives have been taken to use physical activity on prescription (PAP) to encourage physically inactive children to become more active. Previous research shows that interprofessional collaboration plays a crucial role in promoting physical activity in children, as well as in promoting health in schools. However, there is a lack of knowledge about PAP for children in the school setting, including how medical and educational staff can work together to encourage children who have been recommended PAP. Therefore, this study aims to explore the perceived facilitators and barriers concerning interprofessional collaboration regarding physical activity on prescription in the school setting, as viewed from the professionals’ perspectives. Semi-structured interviews were conducted with 21 professionals who work with the method in school settings. The data were analyzed using Reflexive Thematic Analysis. The results reveal both barriers and facilitators for interprofessional collaboration on PAP in the school setting, as perceived by professionals. Organizational and structural obstacles within school institutions hinder collaboration, while a shared commitment to PAP, characterized by consensus-building, acts as a facilitating factor. PAP for children in a school setting is still an unexplored area and further research is required.

## Introduction

Globally, the number of children who meet the WHO's recommendations for physical activity has decreased (1). Physical activity is beneficial as it improves general health and wellbeing (1), as well as cognitive function and academic performance (2). Promoting children's physical activity is thus crucial and needs to take place across diverse societal arenas and levels in society (1). Schools are recognized as a key arena for promoting health and physical activity (1, 3), since they have the potential to reach many children and could thereby reduce social inequalities in health and physical activity (3, 4).

There are many ways to encourage physically inactive children to become more active, and in Swedish school health service (SHS), there are local initiatives to use physical activity on prescription (PAP) (5). Common reasons for recommending PAP to children are high BMI, or self-estimated physical inactivity (ref). PAP is a person-centered and medical approach, originally developed for a health care settings and adult patients with certain diagnoses, providing individuals with a written recommendation for physical activity (6). In adults, PAP has been shown to increase physical activity levels and decrease sedentary time (7). However, knowledge is still limited about the use of PAP with children and about the method's suitability in a school setting. The development of PAP has primarily focused on adults in medical settings. However, it is important to explore the adaptation to children and relevant contexts (8), to establish if and how this method can be used to reach children that would benefit from an increased physical activity level. This can be seen as a step in developing and evaluating a complex intervention (9).

Implementing PAP in the school setting is shown to be a complex and delicate process which needs both competence and organizational resources, as well as internal and external collaboration (5). School nurses, who are often responsible for prescribing PAP in schools, request more interprofessional collaboration to enhance support in the PAP-process and to reinforce their professional role (5). To strengthen the interprofessional collaboration between different professions in the PAP process at school, a supplement to PAP: Co-organized PAP has been introduced (5). This means an expanded collaboration around the PAP model, providing children with a supportive environment in the form of a network of adults—both medical and educational professionals in school and the family. Also in pediatric healthcare, it is shown that collaboration between different professionals and physical activity organizers is essential to respond to children's needs and promoting effective use of PAP (10). Establishing lifelong learning and promoting changes in physical activity patterns is a multifaceted effort. This process necessitates collaboration across diverse arenas, involving professionals from various fields as well as families (1).

Within the general school setting, interprofessional collaboration is seen as beneficial for children's health and well-being (11), and is vital for assisting children with lifestyles changes, such as increasing their level of physical activity (12). Also, the growing complexity of physical and psychosocial health issues among children necessitates early interventions and robust interprofessional collaboration in schools to support both children and their families (13). To handle this complexity, the Swedish Education Act has since 2023 mandated that the work of the School Health Services (SHS) must be carried out in collaboration with teachers and other staff. Moreover, all school staff, based on their roles and competencies, are obligated to contribute to promoting students’ health (14). Interprofessional collaboration is here defined as a collective effort involving two or more professionals from different professional backgrounds, united by common objectives (15).

Although interprofessional collaboration may seem like a solution when implementing new health promotive initiatives, there may be challenges. In the general school setting, research has identified several facilitators and barriers to interprofessional collaboration. Among the facilitators, the importance of strong and supportive leadership from school principals has been emphasized (16). Additionally, certain organizational models have been shown to improve interprofessional team collaboration in schools (17). On the other hand, barriers to interprofessional collaboration include insufficient communication, unclear roles, lack of leadership support and scarcity of time or resources (18, 19). In the Swedish context, SHS prioritizes collaboration (20). However, school nurses have reported ambiguity in role definitions within interprofessional teams (11). The clarification of these roles is essential for effectively addressing children's specific needs (20). A challenge for health professionals working in a pedagogical context, is also the transfer from a medical to a health-promotion and well-being perspective (11).

Currently, there is limited knowledge about PAP to children in the school setting, including how professions can work together to encourage children who have been recommended PAP. Previous research shows that interprofessional collaboration plays a crucial role in promoting physical activity in children, as well as in promoting health in schools. It is therefore valuable to deepen knowledge, not least through medically and pedagogically oriented professionals in schools—who work with children to promote health and well-being. The aim of this study is to explore the perceived facilitators and barriers in the interprofessional collaboration around physical activity on prescription in the school setting, from the professionals’ perspectives.

## Methods

### Study design

To fulfil the study's objectives, a qualitative method was used. This approach is considered appropriate for investigating new domains, enhancing the understanding of a new phenomenon, and capturing individuals’ experiences and perceptions (21). Reflexive Thematic Analysis was employed for data analysis (22). Furthermore, an inductive methodological was adopted (23), allowing for an unbiased textual analysis, such as personal accounts of experiences, in this case perceptions of interprofessional collaboration around PAP.

### Study context

The organization of Swedish schools is overseen by either the municipality organizer or the organizer of the independent school, both of whom are responsible for the education provided in the schoolEach school is in turn governed by a principal, who is responsible for leading local operations, coordinating staff cooperation, and organizing and distributing resources based on the different conditions and needs of the children (14). In Sweden, like many other Western countries, schools are obliged to provide access to SHS, as per the Education Act (24). SHS encompasses medical, psychological, psychosocial, and special pedagogical measures, all aimed at prevention and health promotion to support students’ development toward educational objectives (24).

### Physical activity on prescription

Physical activity on prescription (PAP) is a personalized written prescription for physical activity, structured into three core steps: a person dialogue, an individually tailored physical activity prescription, and a structured individualized follow-up (6). In addition, it includes two supplementary components for methodological support: the evidence-based manual “Physical activity in Prevention and Treatment of Disease” (FYSS), and knowledge support and collaboration with activity organizers. Introduced in Sweden in 2001, PAP is a method used by healthcare providers to promote physical activity for the prevention and treatment of health disorders. All licensed Swedish healthcare professionals with the necessary expertise—such as school nurses, school physiotherapists, school physicians, and school psychologists—are authorized to prescribe PAP within school settings (6). How the need for PAP is assessed differs nationally in Sweden. It is based on local criteria and individual assessments, made by the prescriber in consultation with the individual child and parents. The PAP activity is tailored to the individual child, encompassing everyday exercises organized by an activity organizer, or subsidized activities such as swimming and gym workouts. The person approach allows for variation regarding the type of physical activity and its duration (5).

### Procedure and participants

Participants for the study were selected from four municipalities in southern and central Sweden, each of which implemented PAP in their local schools. These municipalities varied in size—small, medium, and large cities—and encompassed varied geographic areas –rural and urban. The recruitment of participants began with initial contact made via email to four key persons in four municipalities where PAP was implemented in schools. Thereafter, these key persons were asked to inform school staff in their network who used PAP about the opportunity to participate in the study. This was facilitated with the help of a standardized information letter. Inclusion criteria was that the participants worked at schools/municipalities who used PAP. All key persons provided contact details to one people in their municipalities representing a group who used PAP and wanted to participate in the study. Contact with the four groups was made to schedule a focus group interview. To ensure a diverse range of professions and experiences with PAP, a snowball sampling selection was employed. Snowball sampling is a technique often used in scientific research to access susceptible populations (25). In this study, the first author conducted the snowball sampling by asking interview participants if they knew of other professionals with experience using PAP in the school setting. The interview participants suggested four people, who were then asked to participate in the study. All four of these individuals accepted the invitation.

In total, 21 participants were included, representing 10 different professions/roles within the school system: school nurses (*N* = 8), school physicians (*N* = 2), physical education teachers (*N* = 2), principals (*N* = 2), medical management officers (*N* = 2), school physiotherapist (*N* = 1), school curator (*N* = 1), study mentor (*N* = 1), guidance counsellor (*N* = 1), and activity organizer (*N* = 1). The group comprised 16 women and 5 men, each with a range of professional experience spanning from 1.5 to 28 years, with an average of 10.6 years in their respective roles. The participants worked with different age groups within the school system. According to data from the Swedish National Agency for Education (26), these participants were employed at schools with socioeconomic indexes ranging from 39.1 to 137.9. It is important to note that a higher index number indicates a higher level of higher socioeconomic vulnerability. Before the study commenced, all participants provided their informed oral consent to participate.

### Data collection

Data were collected with both focus groups interviews and individual interviews. Focus groups entail a group of individuals who discuss a given subject for a limited time. A focus group method is a suitable way to collect research data from many participants about a specific and pre-determined topic (27). In this study, the focus group method was considered particularly effective for exploring interprofessional collaboration on PAP. However, due to scheduling difficulties and heavy workloads, not all participants could attend the focus group sessions. As a result, these participants were offered individual interviews instead.

A total of five focus groups were formed, with 2–6 participants in each group. In addition, three individual interviews were conducted. The first author conducted these interviews from December 2023 to February 2024. The interviews were carried out either in person or via video conferencing tools such as Zoom or Teams. All sessions were recorded for both image and sound. The duration of the interviews ranged from 26 min to 1 h and 20 min, with an average of 43.36 min. After three interview sessions, the participants received, supplementary follow-up questions, which they responded to in writing. Both the focus group discussions and the individual interviews utilized an open-ended and semi-structured interview guide. The interview guide for the study was developed by the first author and was further tested and evaluated based on feedback from a pilot interview. The interview guide was structured around four thematic areas: current situation description, cooperation and competence, the PAP method, and prospects. Each theme was explored through 2–8 open-ended questions. In addition, the interviews were also supplemented with relevant follow-up questions. After three of the focus group interviews, written follow-up questions were provided for further clarification.

Detailed information about the participants and the interviews can be found in Table 1. The study received ethical approval from the Swedish Ethical Review Authority (Dnr 2022-06577-02).

**Table 1:** Detailed information about the participants and the focus group interviews.

Interview	Gender	Professions/roles	Level	Code name	Forum	Length of interview
1 Focus group	Women	School nurse	High school	Johanna	Teams	40:56 Plus, written answers
1 Focus group	Women	School nurse	High school	Isabella	Teams	40:56 Plus, written answers
1 Focus group	Women	School nurse	Compulsory school	Charlotte	Teams	40:56 Plus, written answers
1 Focus group	Women	School physician	Compulsory school	Mia	Teams	40:56 Plus, written answers
2 Focus group	Women	School physiotherapist	Compulsory school	Emma	Teams	51.26 Plus, written answers
2 Focus group	Women	School nurse	Compulsory school	Sofia	Teams	51.26 Plus, written answers
2 Focus group	Women	School nurse	Compulsory school	Elisabeth	Teams	51.26 Plus, written answers
2 Focus group	Women	School nurse	Compulsory school	Rebecca	Teams	51.26 Plus, written answers
3 Individual	Women	School physiotherapist	Compulsory school	Emma	Teams	26.00
4 Focus group	Man	Physical education teacher	High school	Oscar	Zoom	44.03
4 Focus group	Man	Physical education teacher	High school	Daniel	Zoom	44.03
5 Focus group	Women	School nurse	Compulsory school	Elin	Teams	35.22 Plus, written answers
5 Focus group	Women	Assistant principal	Compulsory school	Julia	Teams	35.22 Plus, written answers
5 Focus group	Women	Principal	Compulsory school	Susanne	Teams	35.22 Plus, written answers
5 Focus group	Women	Guidance counselors	Compulsory school	Marie	Teams	35.22 Plus, written answers
5 Focus group	Women	School curator	Compulsory school	Anna	Teams	35.22 Plus, written answers
5 Focus group	Man	Study mentor	Compulsory school	Johan	Teams	35.22 Plus, written answers
6 Individual	Man	School physician	Compulsory school & High school	Eric	Teams	34.32
7 Individual	Man	Activity organizer	Compulsory school	Anders	Teams	55.31
8 Focus group	Women	Medical management officer	Compulsory school	Ella	Live	1.00.20
8 Focus group	Women	School nurse	Compulsory school	Alice	Live	1.00.20
8 Focus group	Women	Medical management officer	Compulsory school	Eva	Live	1.00.20

### Data analysis

Reflexive Thematic Analysis (RTA) was used to analyse the data (22). RTA emphasizes the active role of the researcher in interpreting data and producing knowledge. This meant that themes werenot only identified and retrieved from the researcher's theoretical assumptions and analytical resources, but also directly from the data itself (22, 28).

The analysis was based on Braun and Clarke's (22) six-stage process, chosen for its flexibility and potential to yield a rich and complex understanding. The first stage involved becoming familiar with the generated data. This was achieved by listening to the recordings, transcribing the interviews verbatim, reading the transcripts multiple times, drafting preliminary analytical notes, and examining the data with an analytical lens to identify recurring meanings and patterns. After familiarization with the dataset, initial codes were generated by systematically coding the entire data in alignment with the research aim and questions. In the third stage, the codes were re-analyzed and then organized into potential themes and sub-themes. This led to the creation of a preliminary thematic map providing an overview of the themes and subthemes. Thereafter, in the fourth stage, these potential themes were adjusted and refined through a recursive process involving the entire dataset and the different stages. This contributed to a rigorous analysis, ensuring that the themes and sub-themes accurately represented the dataset. A refined thematic map was created in this stage. In the fifth stage, the themes were clearly defined and assigned appropriate names. Finally, in the sixth stage, the results section was composed, featuring selected quotes to provide the reader with an understanding of each theme. The quotations, chosen to illustrate the essence of the themes, were translated from Swedish to English. In the discussion, the themes are examined in relation to a theoretical model.

To enhance the trustworthiness of the analysis processes and achieve the reflexivity inherent in the method, the authors employed triangulation (29). The primary responsibility for the analysis was the first author (EW), with the other authors providing reflective comments. Themes and sub-themes were discussed and further adjusted until a consensus was reached among all authors. The authors—with their diverse experiences and backgrounds in physiotherapy, physical education, sport science, interprofessional collaboration, youth health, and public health—contributed unique skills and perspectives. Lastly, to assess the quality of the analysis process, the authors found Braun and Clarke's (23) 15-point “checklist” for a good thematic analysis to be beneficial.

## Results

**Figure 1:**
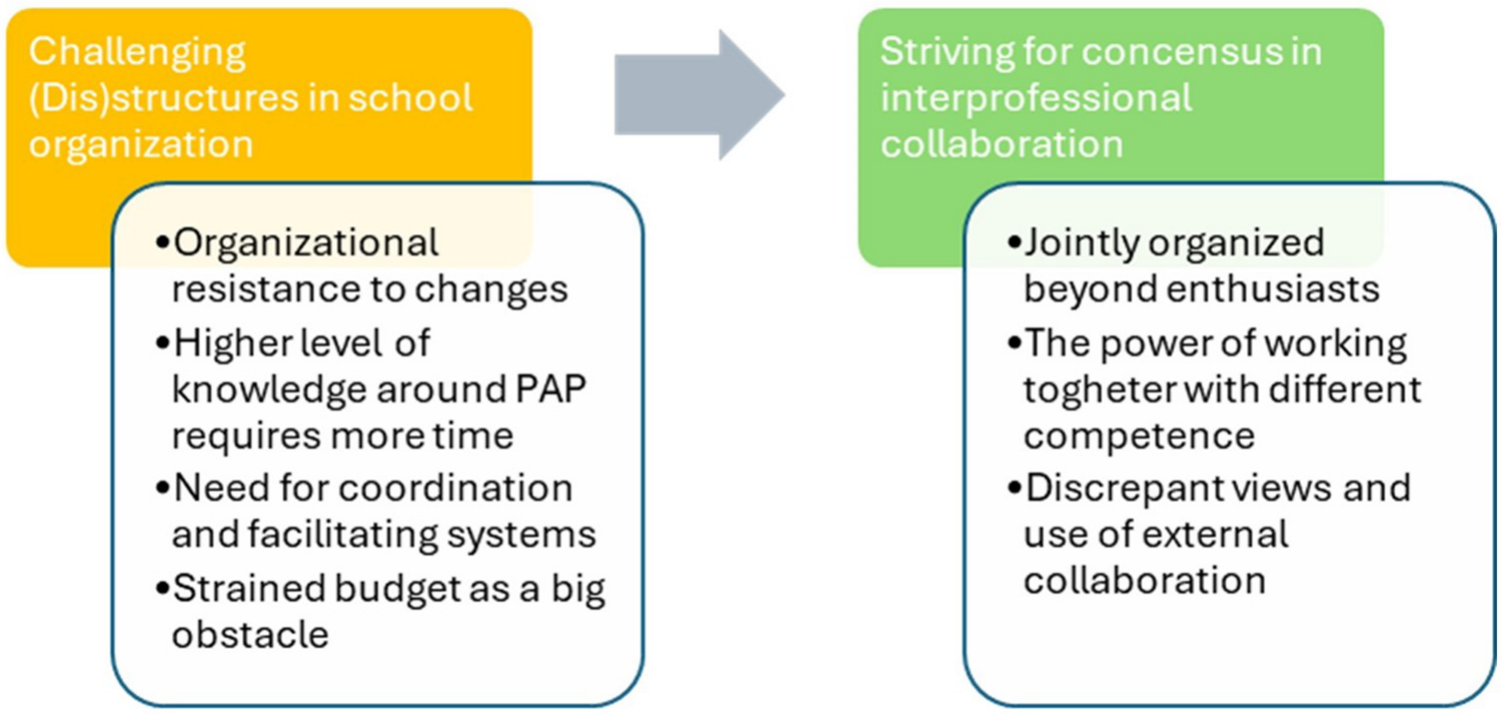
The two themes with sub-themes in relation to each other.

The analysis yielded two themes: “Challenging (Dis)structures in school organization” and “Striving for consensus in interprofessional collaboration,” each with related sub-themes (as detailed in Figure 1). The first theme captures the barriers posed by the presence and absence of structures within the school organization, which impact and challenge the interprofessional collaboration on PAP. The second theme highlights facilitators that contribute to the usefulness and sustainability of interprofessional collaboration on PAP. Collectively, the two themes capture both the facilitators and barriers for interprofessional collaboration on PAP in the school settings. Figure 1 illustrates the relationship between these themes.

### Challenging (Dis)structures in school organization

The sub-theme “Organizational resistance to changes” identifies barriers within the school organization that hinder professional collaboration and implementation of PAP. The adoption of PAP, a novel method that lacks legislative backing, was a slow and inefficient process due to outdated frameworks prevalent within schools:

Emma: I feel there is an organizational resistance [to PAP] because the school is still governed somewhat as it was in the 1800s. You are constantly trying to solve new things through the existing frameworks. I think we need to think anew and dare to look forward.

The school's intricate organizational framework posed barriers to professional collaboration among participants. SHS professionals elaborated on this by emphasizing their frequent encounters with resistance during interprofessional collaboration due to the inherent incompatibility between healthcare and school systems. Furthermore, they expressed feelings, of inferiority, likening their role as having “played as guests in another arena.” This sentiment reflected their experience that their proposals to implement and use PAP in schools were not always met with understanding from the rest of the school staff.

The participants’ desire for broader acceptance of the PAP method within the school connects to the subsequent sub-theme: “Higher level of knowledge around PAP requires more time.” This sub-theme reflects their perceived need for enhanced knowledge of PAP to develop and facilitate sustainable interprofessional collaboration around the method. To increase the knowledge level of the entire school staff, more allocated time was deemed necessary for this work.

The participants reported that school staff observed a concerningly low level of physical activity among many children. Consequently, there was a recognized need for a method within the school system aimed at enhancing children's physical activity levels. However, they felt the work with PAP had initially faced resistance by school staff outside of SHS, primarily due to a lack of knowledge about the PAP method. They highlighted that teachers and other staff outside SHS perceived PAP as an additional workload, leading to a negative outlook. To foster an understanding of PAP and increase the likelihood of staff-wide acceptance of the method, most schools carried out information campaigns targeting all staff. The key message in these campaigns was to clarify the role of the school staff in the PAP process and to underscore that the method did not entail additional work for the teachers:

Johanna: We are very clear with the teachers because they are tired of having something new to do. So, when we are out and talking in the work teams, we are clear that this is no extra work for them.

Oscar: We have been around all the work teams and informed about what PAP means and how it is done, and that their only task is really to be a fellow human being, maybe see signals, dare to sound the alarm if you discover that here it feels like it is not correct. And I don’t think that’s extra work for a teacher because that’s how most teachers already work. If you feel that this child is not feeling well for any reason, then you sound the alarm, I think. You should at least do that. Then you should know that PAP is an alternative method to use in this.

Participants noted that the opportunity to disseminate knowledge about PAP throughout the entire school varied. In some municipalities, there were individuals with the necessary competency, while in others, such expertise was either lacking or missing. Currently, they observed it was primarily physiotherapists, PE teachers, school physicians, or school nurses with an interest in physical activity who contributed to increasing knowledge about PAP. However, these professionals emphasized that the task was time-consuming and challenging to integrate within their regular duties, given that it was not inherently aligned with their primary mission. Despite these challenges, they emphasized the importance of information about PAP. Participants communicated it was simpler to suggest PAP to a child when staff identified a need for it, as opposed to suggesting PAP when the staff did not perceive a need. The participants reflected that a shared understanding of the method among all school staff contributed to the effectiveness of the interprofessional collaboration. Additionally, they underscored the need for continuous education, ideally on an annual basis, to accommodate staff turnover and the integration of new personnel.

In the sub-theme “Need for coordination and facilitating systems,” the participants’ request for more coordination and facilitating systems within the school is outlined. The participants believed that interprofessional collaboration around PAP could be improved with more structed coordination. They noted that the lack of structured working methods led to varied practices across schools. For example, they found it was easier to implement and coordinate PAP in smaller municipalities due to shorter decision-making chains. Furthermore, the lack of structed working methods risked confining the work to a select few with a specific interest in the method. They pointed out that this was not a sustainable structure, as the discontinuation of these individuals in a school could lead to the cessation of PAP usage. Instead, they advocated for the development and coordinated of the method to be a school-wide effort, to be led by the school principal.

The participants identified the development of systems to facilitate the documentation of PAP as a crucial area for improvement, aiming to expedite the documentation process. The importance of having accessible and easy-to-use documentation systems was also highlighted, as these are important for generating statistics and discerning the specific impacts, which are informative for principals and upper management. Presenting statistics to principals was considered valuable, given that each principal oversees the school's operations, including SHS. Consequently, securing the principal's support was considered “super important” and a “foundation.” The participants described their work with PAP as being “very dependent on their principal.” A lack of support and interest resulted in the non-utilization of the method:

Eva: There was a school that wanted to use PAP, but the principal was not interested, and then nothing happened at the time.

The participants observed that the level of support and interest in the PAP method among principals varied across different municipalities. Participants from one municipality reported that principals were receptive to the method, provided it did not affect the school's budget. At the same time, the participants highlighted the importance of principals understanding how the method worked and recognizing the need for support. For example, more time could be set aside for working with the method, and permission could be granted to disseminate information about the method within the school. They believed that such measures could promote interprofessional collaboration on PAP within the school.

Economic constraints as a barrier for interprofessional collaboration were reflected in the final sub-theme: “Strained budget as a big obstacle.” Participants from municipalities facing financial difficulties expressed that the current timing for the implementation of PAP was difficult because the schools were under financial pressure caused by budget cuts. The economic situation was described as “a super big obstacle” to interprofessional collaboration on PAP, which resulted in the deprioritization of its implementation. Major cutbacks in the schools increased staff workload, who had to focus their time on fulfilling the primary statutory core mission, rather than on coordinating and collaborating PAP:

Ella: The economy has meant that all resource persons have been removed from the school. So, it’s in crisis mode in the schools right now. Then you can’t take it. You can barely get the basic assignment together. And then it’s just “not one more thing [PAP]. We cannot!”

Furthermore, due to lack of finances, participants explained how the schools had to restructure their approach to PAP. They were required to operate based on a long-term plan and in phases, given the lack of time and resources to initiate a large-scale project immediately. This resulted in only a few professions being involved in the initial stages of the work. However, they identified facilitating strategies for the long term, which included incorporating PAP into operational plans. This strategy aimed to sustain the method and interprofessional collaboration, even during periods of economic downturn.

### Striving for consensus in interprofessional collaboration

The sub-theme “Jointly organized beyond enthusiasts” highlights the importance of interprofessional collaboration on PAP, as opposed to individual efforts. The participants emphasized that for PAP to be sustainable in the school setting, collaboration across professional boundaries and beyond just the enthusiasts were necessary. They cautioned that a lack of such cooperation could result in PAP being associated solely with specific individuals. This was viewed as an unsustainable structure, as the method risked disappearing from the school if those persons left. Moreover, they highlighted that PAP organized on a personal basis did not promote equality in schools, as the prescription was influenced by the individual's interest and their ability to engage children in physical activity:

Sofia: It depends a lot on certain people at the school, who think PAP is important. And then it becomes very personal; and there is a small clique that works with PAP.

Rebecca: There will be no equivalence.

Sofia: No, there will be no equivalence. It becomes personal that social pedagogue is fun—we can play basketball with her at breaks. But if she hadn’t been a fun social educator, there wouldn’t be any basketball. So, it is very much governed by what kind of person is in the position, as well.

Emma: I’m also saying this is very individual and tied to the individual. If that enthusiast disappears, PAP will fall flat on the ground. Then we don’t have an organization that keeps the structure.

Establishing a stable and sustainable foundation that facilitates PAP goes beyond having enthusiastic people. The participants highlight an advanced development known as GO-PAP. The term GO-PAP signifies that the prescription was jointly organized within the school. It was pointed out that the use of GO-PAP was reinforced by the education law, which stipulates that SHS should be conducted in collaboration with the rest of the school:

Ella: The school must work in a structured way with SHS, and you should develop collaboration and get it into your quality system. And both of those things are GO-PAP.

The participants observed that the adoption of GO-PAP varied across the municipalities. The focus was primarily on collaboration within SHS, notably among school nurses, school physicians, and physiotherapists. However, they highlighted the importance of including more professions both within and beyond to facilitate PAP. Participants from one school shared their successful experience with GO-PAP, having formed a local group that consisted of PE teachers and school nurses. This group reported favorable outcomes, noting that the involvement of diverse professions contributed to the development and support of a structured approach:

Johanna: I recommend a local GO-PAP group because then you are not so alone, but you have the support of the PE teachers. I worked at another school in another municipality, and there we had the same thing. But that also included a social worker who was out at the schools and met the students a lot. So, I think it might look a little different; it’s a matter of taste. But I think it works well.

There was a shared understanding that collaboration between SHS and PE teachers was beneficial, yet barriers existed. As per the school nurses, these barriers arose because the PE teachers did not have time to get involved in PAP:

Sofia: One would have wished it had been better. It’s a locked position there. They [PE teachers] look after their physical education lessons, and that is their mission. I don't think they will become any GO-PAP people, to be completely honest.

Rebecca: Unfortunately, neither do I.

Sofia: No, it is if I’m going to be crass. But maybe an individual, but it will be difficult for an individual to run it. So, at group level for the physical education teachers, they stick to their physical education lessons and their plan. So, it is difficult to reach there.

Emma: I'm a bit surprised by the resistance from the physical education teacher group, as a large group. There is no one who spontaneously says “Yes, please come, we have a collaboration.” But it’s a lot of prestige, it feels like. Then there are individuals who are different, I must say that. But in a large group it is much more difficult.

The second sub-theme, “The power of working together with different competence,” highlights the diverse professionals within the school context and their potential role in the PAP process. The participants recognized the significant potential and strength in utilizing PAP within the school setting, as different professions offer specific and unique professional competencies. One physiotherapist likened it to a strength when there are ‘more puzzle pieces to complete the puzzle.’ Furthermore, the participants highlighted that those different professions possessed unique competencies related to physical activity, relationships, and time spent with children. The diversity implies that different professionals had different opportunities to identify children who needed PAP. While only licensed health professionals in SHS are authorized to prescribe PAP, the involvement of other professionals in identifying children in need of PAP is crucial. By distributing the responsibility, a larger number of children could be provided with PAP.

Although the participants acknowledged the importance of all professions working with PAP and regarded the methods as an “alternative in their toolbox,” certain professions were singled out as key contributors. Physiotherapists and PE teachers were distinguished for their inherent professional skills in promoting and adapting children's physical activity. School nurses were also highlighted for their role in discussing physical activity with all children during health dialogues. For younger children, professionals who spent a significant amount of time with them, such as teachers, leisure staff, and resource persons, were deemed crucial in the PAP-process:

Rebecca: When we get started with GO-PAP, I think there will be a lot of recreational staff or resource staff that will be important. They are usually the ones who are closest to the child and who have another opportunity to follow the child more continuously during the school day, because they are often in the classrooms and during free time, throughout the school day.

Elisabeth: Especially in primary and intermediate, where the teachers are so much with their class. They know everything about their students, after all.

For older children and adolescents, mentors, counsellors, and psychologists were highlighted as key persons. Their one-to-one conversations with the children provided valuable insights into each child's situation. Furthermore, as PAP aimed for changing lifestyle habits related to physical activity, the inclusion of counsellors’ and psychologists’ experience in motivational conversations is crucial for success:

Emma: I think psychologists should be able to prescribe PAP. They can see the connection between inactivity and mental illness.

The participants identified professions that facilitated PAP, but they also acknowledged barriers preventing the full utilization of professionals’ skills. These barriers could be due to limited or no access to certain professions, for example physiotherapists, or that not all PE teachers interacted with students across all grades. Another constraint was the extensive responsibilities of school physicians, who oversaw many students, leaving them with insufficient time to prescribe PAP.

The concluding sub-theme, “Discrepant views and use of external collaboration,” reflects the participants’ views on cooperation beyond the school setting. Healthcare entities, sports organizations, and activity organizers served as key collaborators. The participants detailed a large variety of approaches in engaging these external partners and underscored their role in facilitating the PAP process.

Concerning collaboration with local activity organizers, certain schools have developed partnerships that facilitated subsidized PAP activities like swimming and gym. An activity organizer, experienced in hosting GO-PAP children for several years, has proven to be an effective facilitator in the PAP process. They were an important link between the school and club sports, offering support and guidance to children who received PAP. The collaboration with municipality schools was robust, with regular school visits to promote activities. However, the activity organizer seeks to be more involved in the PAP process, aspiring for more children with PAP to join their activities and a more defined role in the follow-up process:

Anders: We would have liked to see the school nurses send more school children with PAP to us. Or that they had done it in a different way and developed it a little more. But it works fine as it is right now. We are more than happy to join.

To facilitate the children who received PAP, some municipalities coordinated their range of activities with local organizers to compile an activity catalogue. The participants from one municipality reported that they sought help from another municipality in another region to draw inspiration for their own catalogue:

Eric: We are making an activity catalogue, largely based on the model from Skåne [region], and this GO-PAP. So, we have been given permission to look at their activity catalogue, and we will soon have our own.

Moreover, they sought help from RF-SISU, which served as a “link between school and association life.” This approach facilitated the creation of connections with association sports, which could then be included as a proposal in the activity catalogue. A school doctor pointed out the importance of establishing contact with associations that offered activities tailored to children who were not used to physical activity or lacked experience of association sports:

Eric: We have tried to stay with the organizations that have activities that we think are suitable for those who are not used to physical activity. We have a few football organizations, but that’s not what children and adolescents choose. I think that team sports, such as football, floorball, ice hockey and the like, are a bigger step to take. We have other team sports like underwater rugby, which I think is much more inclusive. The same thing with shooting and archery, there we have associations that are very good at including those who are far from association life. I think it is easier to choose the smaller sports.

At the same time, some participants expressed no requirement for an activity catalogue nor external actors. They felt that there were risks in relying solely on an activity directory, as not all children were prepared for activities they offered. Instead, they advocated for “low threshold” activities, which could be self-directed:

Elisabeth: The prescriptions that I have prescribed have been from a very low level, almost zero. So, there it has been very basic. It’s about just going out for a walk with your mother or starting to cycle to school. So, I think that’s enough.

Sofia: I agree with you on that. I’ve written some PAP where the boy wanted to go to the gym, but otherwise ones I’ve written have been…

Elisabeth: get up off the couch.

Sofia: Yes, to break sedentary. So, it has been low threshold like that.

Healthcare was seen as another facilitating collaboration partner for schools. Some participants shared positive experiences of working with healthcare professionals, where school nurses could refer children to receive PAP. There is a desire among participants to initiate more cooperation with child and adolescent psychiatry, children and youth rehabilitation services, and social services. Furthermore, a municipality has begun a partnership with a primary healthcare center, allowing the school's SHS team to refer children who would benefit from PAP consultations. These children can then engage in PAP at the health center's gym, supported by physiotherapists, primarily aiding those with additional support needs who may struggle in other social contexts or organized club sports:

Ella: It’s the rehab clinics on the primary healthcare center that we get to work with. If we get a child who we feel needs more support from a physiotherapist, then we can send them there. So, then they start training there. And the health center is also positive. If we have many children with neuropsychiatric disorders problems, then they could have a small PAP group for them. It’s nice if you take children from different schools, so that they can meet and socialize in the right way.

Eva: It’s a win for society in the end. Those are huge profits. Both financially and for the individual child.

Ella: And just when it was neuropsychiatric disorders that we thought a lot about, it feels extra urgent to start on that end.

In summary, the first theme primarily indicates barriers to the interprofessional collaboration around PAP within the school setting. Conversely, the second theme primarily captures the factors that facilitate such collaboration.

## Discussion

This study highlights the dynamics of interprofessional collaboration on PAP in school settings, underscoring professionals’ views on the facilitators and barriers to collaboration within these contexts. The perceived challenging (Dis)structures present in school organizations are seen as barriers to interprofessional collaboration. In contrast, a perceived shared commitment to PAP, characterized by a collective pursuit of consensus, emerges as a facilitating factor. These results mirror established knowledge from implementation research, showing the importance of functioning organizational models (17), supportive leadership (16), and shared goals among professionals (30), when implementing new interventions. Our results also indicate that insufficient communication and unclear role definitions impede interprofessional collaboration when implementing PAP in school, a conclusion consistent with previous research on interprofessional collaboration (18, 19).

Within the school context examined in this study, professionals express diverse and sometimes discrepant views regarding the value of physical activity programs. These differing perspectives directly impact the conditions for interprofessional collaboration. Professionals within the SHS noted that it was primarily non-SHS professionals who questioned the need for PAP. Consequently, SHS staff emphasized the necessity for widespread knowledge about PAP throughout the school. This observation aligns with implementation research, which underscores the need for potential users to understand and value a method for successful implementation (31). Since PAP originates from a medical paradigm, it is not surprising that SHS staff (with medical education) see the advantages with PAP, compared to non-SHS professionals, without a medical perspective. Professionals with a pedagogical responsibility may feel that physical activity promotion is outside their assignment and increase the already heavy workload (32).

While schools have the potential to reach all children and are thus well-suited for implementing PAP, this strategy is not without risks. As indicated by the professionals in our study, there exists a risk of unnecessary medicalization or stigmatization of individual children or specific groups when implementing PAP within the school context. A fear of stigmatizing children who received PAP is also demonstrated in previous research about PAP in schools (5). Unlike collectively oriented strategies, such as those found in Physical Education or in the framework of health promoting schools (33), which encompass all students, PAP specifically targets children deemed ‘at risk’. The potential “clash” between a medical and educational paradigm in the implementation of health promotion interventions in schools, as well as issues of potential medicalization by a “medical gaze” on children, are crucial to explore further in additional national and international contexts.

Furthermore, the participants perceived that the Swedish school system lacks established structures and frameworks for interprofessional collaboration, which affects their opportunities to develop PAP. They perceived a lack of guidelines and structures as significant barriers at the organizational levels. This concern is mirrored by the Swedish National Agency for Special Needs Education and Schools, which problematizes the absence of guidelines for interprofessional collaboration in schools in their report about sustainable school health work (34). Moreover, our results suggest that the absence of well-defined structural frameworks affects collaboration. The lack of specific guidelines leads to variability in how local collaborations concerning physical activity programs (PAP) operate. At the same time, it is not surprising that national guidelines for PAP in the school context are lacking, since PAP in this context is relatively new and unexplored. Guidelines and recommendations for PAP for adults are based on solid evidence for the method. In relation to PAP for children, more research is therefore needed in the school setting, before guidelines can be designed. This procedure can be likened to that of the development and evaluation of complex interventions (9), which, as in the current study, may imply adapting an existing intervention to a new population or setting. When adapting and refining an intervention, it is crucial to involve stakeholders and to address key-uncertainties and contextual conditions (9). By doing this, the current study contributes to the process of evidence generation, for PAP in a new context (school setting) to a new population (children).

In relation to the fact that PAP is still an unexplored method for children in school, the results in this study raise an important question: Who bears responsibility for children's physical activity? Given the complexity of children's activity and the influence of social determinants (35, 36), it is clear that schools alone cannot be the sole solution. Several actors in society need to be involved and work together, including civil society, sports clubs, and parents (1). Despite schools having an increasingly important role in supporting children to become more physically active (37), the lack of specific guidelines defining this role may hinder interprofessional collaboration on PAP, as there is no clear sense of responsibility. This situation may be linked to “professional ethnocentrism” (38), where professionals only view the reality from the lens of their own field (39), thereby potentially overlooking the broader needs of children's physical activity. At the same time, the results highlight typical challenges with introducing new methods in rigid and reluctant organizations, according to Rogers’ diffusion of innovation theory (39). People's adoption of new methods does not happen simultaneously in a social system; rather it is a process whereby some people are more apt to adopt the method than others. From this theory the participants in this study can be seen as “innovators” and some “early adopters”, who want to convince the “early majority” about PAP. The key to adoption is that the person must perceive the method as new or innovative. Linked to the results in this study, barriers in interprofessional collaboration about PAP can be due to people not seeing a need for the method and therefore no adaptation takes place. From a research perspective, PAP needs more evaluation before implemented on a larger scale.

To note, a significant result in this study is that a robust local platform or core can mitigate the absence of frameworks. The participants advocate a further development of PAP, GO-PAP, a jointly organized PAP initiative at schools that provides a stable and sustainable basis for the work. This approach is supported by the education law that mandates collaborative SHS-work within schools, and it aligns with research advocating the importance of multi-interventions involving various actors for effective health promotion (13, 40). Furthermore, the study identified the pivotal role of school principal support as a facilitator for interprofessional collaboration on PAP. The results suggest that schools with supportive principals are better equipped to establish their own PAP guidelines at local level, compensating for the lack of clear guidelines at national level. This aligns with the findings of Borg and Pålshaugen (41), who have shown that the absence of standardized guidelines provides an opportunity for local initiatives to enhance collaboration among various professions according to local requirements. In the context of implementation research, there is a strong emphasis on local leadership and the need to tailor strategies to specific local conditions. This approach ensures that standard procedures are seamlessly integrated into the unique context, thereby promoting successful implementation (31, 42).

Our study indicates that local conditions influence how external actors were perceived and utilized within the PAP process. Certain participants expressed a need for collaboration with activity organizers, healthcare centers, and organized sports clubs. However, others did not seek such cooperation, as children expressed a preference for their own activities. Notably, PAP is an individualized prescription for physical activity, and a prior study has emphasized the importance of tailoring PAP to each child's unique needs (5). Additionally, research confirms that children engage in more physical activity when they participate in enjoyable activities (43). Therefore, external collaboration for PAP should be guided by the child's interest and needs rather than on local availability. However, this study suggests that local conditions do create diverse possibilities for such activities.

The findings underscore the intricacy of implementing and collaborating around PAP within the school context, influenced by divergent perspectives on PAP and the complex organizational structures within schools. To further highlight the structural challenges and critically examine perceived facilitators and barriers related to PAP in schools, it is useful to discuss the results in the context of Bronfenbrenner's bioecological theory (44–47). The theory provides an overall holistic perspective (46), based on four system levels—micro, meso, exo, macro—that interact with each other in complex patterns. The model aids in understanding how these levels influence the opportunities (facilitators and barriers) for professional collaboration in schools regarding the PAP method. The exosystem levels pertain to external institutions and factors that impact school operations. At the macro system level, we consider the broader cultural and ideological context within society [as proposed by Bronfenbrenner (44); Bronfenbrenner and Morris (45)]. This context indirectly influences interprofessional collaboration concerning PAP. Our study reveals that at the mesosystem level, collaboration around children's physical activity and PAP within schools faces challenges related to interprofessional dynamics. The macrosystem, in our study, may encompass factors like stigmatization and medicalization, rooted in societal norms of what is considered “normal” or “healthy”. Although professionals at the mesosystem level cannot directly alter these views, awareness and acknowledgment remain essential to address these issues. Situating our study within Bronfenbrenner's systems theory, could promote clarity into the complex structures. For PAP to be beneficial for the children, implementation requires engagement from actors across all system levels in the bioecological model.

Taken together, this study contributes to our understanding that collaborative PAP initiatives, such GO-PAP, are perceived as valuable in the school setting among professionals who work with them on a daily basis. This besides challenges and issues in need to problematize. This insight is important for future consideration of PAP implementations in school settings, both nationally and internationally. Future research from diverse international contexts could offer additional perspectives, potentially examining, for example, the roles of various professionals and educational systems, as perceptions may vary based on these aspects. At the same time, PAP for children is still an unexplored area, and more research is needed to understand the feasibility and acceptability of the PAP method in the school setting. Future studies need to explore PAP from the perspective of children themselves, as well as issues of potential stigmatization or medicalization.

### Strengths and limitations

A strength of this study was the chosen qualitative approach, particularly given its suitability for researching new phenomena (21). The pragmatic combination of focus groups and individual interviews, both face-to-face and digital, can be seen as a strength as it facilitated participation. It can also be viewed as a form of qualitative triangulation by the use of different methods (48). This approach contributed to a broad and comprehensive understanding of the topic (48, 49). Another strength lies in the relatively large and heterogenous sample, which varied in terms of professions, schools, children's age, municipalities, and geographic areas with socioeconomic indexes. This diversity provided a rich base for analytical transferability to other similar settings. Validity and trustworthiness were further enhanced by data triangulation, conducted by researchers from different backgrounds and competence areas.

The study also has certain limitations in its methodology. The recruitment process, which relied on key persons, might have inadvertently excluded more critical voices, although it did help in engaging a diverse group of professionals with varying insights. The use of snowball sampling (50) may have resulted in some participants with similar experiences and beliefs, but this was mitigated through seeking professionals with varied experiences. Additionally, the choice of conducting the interviews either digitally or in person may have influenced the spontaneity and depth of the discussions. However, allowing participants to choose their preferred interview format and the convenience of digital interviews likely contributed positively to the study, as supported by other studies using digital interviews (51).

## Conclusion

This study has identified both barriers and facilitators for interprofessional collaboration on PAP in the school setting, as perceived by both medically and pedagogically oriented professionals. Taken together, this study shows that challenging (Dis)structures present in school organizations are perceived as barriers to interprofessional collaboration. Moreover, the results indicate that insufficient communication and unclear role definitions impede interprofessional collaboration when implementing PAP in school. In contrast, a perceived shared commitment to PAP, characterized by a collective pursuit of consensus, emerges as a facilitating factor. Finally, it is important to point out that this study shows various opinions about PAP in the school setting, between SHS professionals and non-SHS professionals. PAP for children in a school setting is still an unexplored area. More research of PAP in the school setting is needed—including children's perspectives, to understand the feasibility and acceptability of the PAP method in this context.

## Data Availability

The original contributions presented in the study are included in the article/Supplementary Material, further inquiries can be directed to the corresponding author.
